# YTHDF1 promotes mRNA degradation via YTHDF1‐AGO2 interaction and phase separation

**DOI:** 10.1111/cpr.13157

**Published:** 2021-11-25

**Authors:** Jiong Li, Ke Chen, Xin Dong, Yating Xu, Qi Sun, Honghong Wang, Zhen Chen, Cong Liu, Rong Liu, Zhe Yang, Xiangfei Mei, Rongyu Zhang, Liuping Chang, Zongwen Tian, Jianjun Chen, Kaiwei Liang, Chunjiang He, Mengcheng Luo

**Affiliations:** ^1^ School of Basic Medical Sciences Wuhan University Wuhan China; ^2^ Hubei Provincial Key Laboratory of Developmentally Originated Disease Wuhan China; ^3^ Department of Urology Tongji Hospital Tongji Medical College Huazhong University of Science and Technology Wuhan China; ^4^ College of Life Science Liaoning University Liaoning China; ^5^ College of Biomedicine and Health Huazhong Agricultural University Wuhan China; ^6^ Department of Systems Biology City of Hope Comprehensive Cancer Center Los Angeles California USA

**Keywords:** AGO2, LLPS, mRNA degradation, RNA patchs, YTHDF1

## Abstract

**Objectives:**

YTHDF1 is known as a m^6^A reader protein, and many researches of YTHDF1 focused on the regulation of mRNA translation efficiency. However, YTHDF1 is also related to RNA degradation, but how YTHDF1 regulates mRNA degradation is indefinite. Liquid‐liquid phase separation (LLPS) underlies the formation of membraneless compartments in mammal cells, and there are few reports focused on the correlation of RNA degradation with LLPS. In this research, we focused on the mechanism of YTHDF1 degraded mRNA through LLPS.

**Materials and Methods:**

The CRISPR/Cas9 knock out system was used to establish the *YTHDF1* knock out (*YTHDF1*‐*KO*) cell lines (HEK293 and HeLa) and *METTL14* knock out (*METTL14*‐*KO*) cell line (HEK293). 4SU‐TT‐seq was used to check the half‐life changes of mRNAs. Actinomycin D and qPCR were used to test the half‐life changes of individual mRNA. RNA was stained with SYTO RNA‐select dye in wild type (WT) and *YTHDF1*‐*KO HeLa* cell lines. Co‐localization of YTHDF1 and AGO2 was identified by immunofluorescence. The interaction domain of YTHDF1 and AGO2 was identified by western blot. Phase separation of YTHDF1 was performed *in vitro* and *in vivo*. Fluorescence recovery after photobleaching (FRAP) was performed on droplets as an assessment of their liquidity.

**Results:**

In this research, we found that deletion of *YTHDF1* led to massive RNA patches deposited in cytoplasm. The results of 4SU‐TT‐seq showed that deletion of *YTHDF1* would prolong the half‐life of mRNAs. Immunofluorescence data showed that YTHDF1 and AGO2 could co‐localize in P‐body, and Co‐IP results showed that YTHDF1 could interact with AGO2 through YT521‐B homology (YTH) domain. We confirmed that YTHDF1 could undergo phase separation *in vitro* and *in vivo*, and compared with AGO2, YTHDF1 was more important in P‐body formation. The FRAP results showed that liquid AGO2 droplets would convert to gel/solid when *YTHDF1* was deleted. As AGO2 plays important roles in miRISCs, we also found that miRNA‐mediate mRNA degradation is related to YTHDF1.

**Conclusions:**

YTHDF1 recruits AGO2 through the YTH domain. YTHDF1 degrades targeting mRNAs by promoting P‐body formation through LLPS. The deletion of *YTHDF1* causes the P‐body to change from liquid droplets to gel/solid droplets, and form AGO2/RNA patches, resulting in a degradation delay of mRNAs. These findings reveal a previously unrecognized crosstalk between YTHDF1 and AGO2, raising a new sight of mRNA post‐transcriptional regulation by YTHDF1.

## Graphical abstract

Working model: YTHDF1 interacts with AGO2 (AGO2 perhaps along with miRNA) through YTH domain, then undergoes LLPS to aggregate more components of miRISCs, and leading to P‐body formation for mRNA degradation. Deficiency of YTHDF1 disrupts the interaction between YTHDF1 and AGO2, leads to the conversion of AGO2 liquid droplets to gels/solids and substantially increases the mRNA stability.

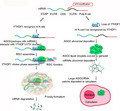

## 1 INTRODUCTION

*N* ^6^‐methyladenosine (m^6^A) is the most prevalent internal messenger RNA (mRNA) modification and regulates stability of targeting mRNAs.^1^ The m^6^A methylation in mRNAs is mainly installed by a writer complex composed of METTL3 and METTL14^2^, ^3^ in a sequence context of RRACH (R = A or G; H = A, C, or U).^3^ The m^6^A reader protein utilizes different mechanisms to recognize and bind to m^6^A‐containing RNAs. For example, the YTH‐domain‐containing proteins (YTHDF1/2/3 and YTHDC1/2) use YTH domain to recognize m^6^A methylation.^1^ Cytoplasmic YTHDF2 promotes degradation of its targeting transcripts partially through recruiting the CCR4‐NOT deadenylase complex.^4^, ^5^ By contrast, the other two cytoplasmic m^6^A readers, YTHDF1 and YTHDF3, are suggested to promote translation of targeting transcripts in HeLa cells by recruiting translation initiation factors.^6^, ^7^ Recent researches showed that the combined activity of YTHDF1/2/3 proteins leads to degradation of m^6^A‐modified mRNA.^8^

When solutions of macromolecules such as proteins or nucleic acids undergo LLPS, they condense into a dense phase that often resembles liquid droplets, and this dense phase coexists with a dilute phase. The driving force underlying LLPS is the exchange of macromolecule/water interactions for macromolecule/macromolecule and water/water interactions under conditions for which this process is energetically favorable.^9^, ^10^, ^11^, ^12^ Importantly, whether a solution undergoes phase separation depends strongly on the concentration and identities the macromolecules and the solution and also the environmental conditions including temperature, salt type, and concentration, co‐solutes, pH, and the volume excluded by other macromolecules.^12^, ^13^ The physicochemical properties of LLPS suggest a variety of possible functions of phase separation processes in cells, such as transcriptional or translational programs,^13^, ^14^, ^15^ RNA metabolism,^16^ and signal transduction.^17^ LLPS can locally concentrate molecules in condensates to activate reactions, signaling processes, and nucleation of cytoskeletal structures.^16^, ^18^ Increasing the local concentration of a key enzyme or protein complex in a condensate could accelerate biochemical reactions.^18^

The miRISCs contain the Argonaute (AGO) family protein AGO2, which plays important roles in the function of miRNA.^19^, ^20^, ^21^ Previous studies have reported the localization of miRISC components to the P‐body,^19^ during this process, GW182 can interact with AGO2 to form P‐body.^22^, ^23^, ^24^, ^25^ P‐body is a membrane‐free organelle and is implicated in post‐transcriptional regulation of mRNAs.^26^, ^27^ Mounting evidences have shown that liquid‐liquid phase separation (LLPS) underlies the formation of membraneless structures.^18^ However, the mechanism of phase separation in RNA degradation has yet to be understood.

Here, we found that YTHDF1 could promote mRNA degradation. Mechanistically, YTHDF1 could recruit AGO2 through the YTH domain, and YTHDF1 played important roles in P‐body formation. Deletion of *YTHDF1* led to P‐body from liquid droplets convert to gel/solid, massive RNA patches deposited in the cytoplasm and delay mRNAs degradation.

## 2 MATERIALS AND METHODS

### 2.1 Data collection

Raw data of CLIP‐Seq datasets for AGO2 and YTHDF1 were downloaded, HEK293:AGO2 (SBDH118, GSM1065670; SBDH28, GSM545213; SBDH29, GSM545216, SBDH30, GSM545217; SBDH40, GSM714642; SBDH41, GSM714643; SBDH43, GSM714644; SBDH44, GSM714645; SBDH45, GSM714646; SBDH46, GSM714647), YTHDF1 (POSTAR2, GSM2064705), HeLa: AGO2 (SBDH104, GSM1048187), YTHDF1 (SBDH207, GSM1553242). And one meRIP‐seq (m^6^A‐Seq) dataset in HEK293 was acquired from GEO database (GSM1339401 and GSM1339402).

### 2.2 Distribution of m^6^A and RBP peaks

MetaPlotR^28^ was used to identify the distribution of m^6^A peaks and RBP binding sites. Gene annotation (GENCODE v31) was downloaded from UCSC genome browser (http://genome.ucsc.edu/). The genomic coordinates of m^6^A and RBP peaks from STARBASE were converted to hg38 via LiftOver (http://genome.ucsc.edu/cgi‐bin/hgLiftOver).

### 2.3 Analysis of m^6^A reader binding

We obtained the CLIP‐seq data of m^6^A readers (IGF2BP1, IGF2BP2, IGF2BP3, YTHDF1, YTHDF2, and YTHDF3) from POSTAR2 database. Peaks were annotated with ChIPseeker (v1.5.1).^29^ Genes with m^6^A peaks in the 3’ UTR region and genes with AGO2 binding were overlapped and selected as the candidate gene for further analysis.

### 2.4 Gene enrichment analysis

We selected genes that have at least 1.5 times prolonged half‐life times in *YTHDF1* deleted cells and performed gene enrichment through the Metascape website (http://metascape.org/gp/index.html#/main/step1). Top 20 significant categories were selected and displayed using the ggplot2 package.^30^

### 2.5 Cell culture and transfection

The Human Embryonic Kidney 293 cell line (HEK293) and HeLa cell line were cultured in DMEM medium (11965092, Gibco™) supplemented with 10% fetal bovine serum (S711‐001S, Lonsera) and 1% penicillin‐streptomycin (10378016, Gibco™). All cell lines were routinely tested for mycoplasma contamination. Plasmids were transfected into cells with Lipofectamine 3000 reagent (L3000150, Invitrogen) according to the manufacturer's protocol.

### 2.6 Immunofluorescence Staining

Cells were grown on acid‐treated glass coverslips. Treated cells were fixed with ice‐cold 4% paraformaldehyde (MKCB4217, sigma) for 20 min, washed 1–3 times with phosphate‐buffered saline (PBS), and permeabilized with 0.5% Triton X‐100 in PBS for 20 min. After washed 1 to 3 times with 0.05% Tween‐20 in PBS, the samples were blocked in PBS containing 1–2% BSA for 1 h at room temperature. Cells were incubated with primary antibody for 16–24 h at 4°C. After washed 1–3 times with 0.05% Tween‐20 in PBS, and secondary antibody for 1–2 h at 37°C. The slides were washed three times with 0.05% Tween‐20 in PBS and sealed the slides with DAPI. Finally, observed under a fluorescence microscope. Images were analyzed using the Image J software. The antibodies used in this research are listed in Table S1.

### 2.7 Co‐immunoprecipitation

Cells were grown in dishes at 70–80% confluence and were lysed with NP40 buffer (150 mM NaCl, 1.5 mM MgCl_2_, 0.5% NP40, TritonX‐100 1%, 50 mM Tris‐HCl at pH 8.0). Proteins were immunoprecipitated from cell lysates with antibody and the corresponding IgG (as described above, AC011, Abclonal). Antibodies were bound to Protein A/G Magnetic Beads, after immunoprecipitation, the beads were washed 3 to 6 times with NP40 buffer. Finally, analyzed by western blotting. FLAG‐tag proteins were purified by Flag‐Beads (IP0064, DianAn Biotech).

### 2.8 Western blotting

Cells were lysed using RIPA buffer (Tris 20 mM, NaCl 150 mM, KCl 20 mM, Mgcl_2_ 1.5 mM, glycerol 10%, Triton X‐100 1%, PH 7.5), and the protein concentration was measured by BCA Protein Assay Kit (C503021; Sangon Biotech). Equal amounts of proteins were separated by 10%–12% sodium dodecyl sulfate‐polyacrylamide gel electrophoresis and were transferred onto polyvinylidene fluoride membranes, and the target proteins were finally detected using standard western blotting protocols and visualized using the Super Signal West Pico Plus Luminol/Enhancer Solution (UC279012, Thermo). Detailed information of primary antibodies and secondary antibodies are listed in Table S1.

### 2.9 RNA staining

The labeling solution consists of a 500 nM solution of RNA Select green fluorescent cell stain (SYTO™ RNASelect™ Green Fluorescent cell Stain‐5 mM Solution in DMSO, S32703, Thermo) in cell‐culture medium appropriate for the cells being stained or phosphate‐buffered saline (PBS). To prepare 1 ml of the labeling solution, a 5 μM intermediate stock was made by adding 1 μl of the 5 mM stock solution to 1.0 ml of the medium and mixed. The intermediate stock (100 μl) was added to 900 μl of medium. The labeling solution (500 nM) was prewarmed at 37°C prior to application and used immediately. A sufficient amount of the prewarmed 500 nM labeling solution was applied to cover the cells adhered to a coverslip and incubated for 20 min at 37°C. When labeling was completed, the labeling solution was removed; cells were rinsed twice in cell‐culture medium or PBS and rested for 5 min in medium at 37°C ready for imaging.

### 2.10 Knockout cell line establishment

We used the lentiviral CRISPR/Cas9 system to establish knockout cell lines. After ligation of T4 DNA Ligase (EL0014, Thermo Scientific™), the ligated plasmid was transformed into *E. coli*. LentiCRISPRv2, and packaging plasmids pMD2.G and psPAX2 were co‐transfected into HEK293T cells, and viral supernatant was collected after 48‐hour transfection. The lentivirus was used to infect targeting cells, which were screened at least 72 h with puromycin after infection. Monoclonal cells were picked for culture by limiting dilution. Western blot and genome sequencing were used to verify knockout efficiency. Genome sequencing primer: YTHDF1‐sg‐F: CACACATAAGTCTTCTTAGAC, YTHDF1‐sg‐R: ACACACACACCTCCACTG.

CGT, METTL14‐sg‐F: TCTACTGAGGAAAGCTATGAG, METTL14‐sg‐R: AGAATCTAAAATTTACACTCA.

### 2.11 Construction of stable overexpressing cell lines

We used pCDH‐CMV‐AGO2‐coGFP and pLVX‐EF1α‐YTHDF1‐mCherry to package virus for targeting cell infection. Monoclonal cells for culture were picked for culture by limiting dilution. Validated by fluorescence to ensure successful construction.

### 2.12 Stranded‐specific mRNA sequencing

Total RNA was extracted with Trizol (Invitrogen) following the manufactory's instruction. The extracted RNA was digested with DNase I (NEB) to remove the genomic DNA before RNA cleanup. About 1 μg total RNA was spiked‐in with S2 RNA and used for mRNA‐seq library preparation. Briefly, mRNA was purified with VAHTS mRNA capture beads, and reverse transcription was performed with NEB ProtoScriptII and a random hexamer oligo (HZG883: CCT TGG CAC CCG AGA ATT CCA NNN NNN). After reverse transcription, RNA was removed by RNase A and RNase H treatment. The single‐stranded cDNA was then ligated with a partial Illumina adaptor (HZG885:/5phos/AGA TCG GAA GAG CGT CGT GTA GGG AAA GAG TGT ddC) using NEB T4 RNA ligase 1 through incubation overnight at 22°C. After SPRI beads purification, ligated cDNA is amplified by 12 cycles of PCR using oligos that contain full Illumina adaptors (LC056: AAT GAT ACG GCG ACC ACC GAG ATC TAC ACT CTT TCC CTA CAC GAC GCT CTT CCG ATC T and Index primer: CAA GCA GAA GAC GGC ATA CGA GAT <index> GTG ACT GGA GTT CCT TGG CAC CCG AGA ATT CCA). Indexed cDNA libraries were pooled, and pair‐end sequenced on an Illumina NovaSeq 6000. Reads were mapped to the human transcriptome (ensemble 96) using HISAT2 allowing three mismatches. The stranded‐specific track files were generated with deeptools 3.3.0 and viewed on UCSC genome browser. The read counts across each gene were counted with HT‐seq with the union mode, and Deseq2 was used to analyze the differentially expressed genes. The heatmap was generated by R package 3.4.3 with normalized counts from Deseq2.

### 2.13 Transient transcriptome sequencing (TT‐seq)

About 1 × 10^7^ cells were labeled with 200 µM 4‐thiouridine (4SU) in a CO_2_ incubator at 37°C for 60 min and were quickly lysed with 4 ml Trizol (Invitrogen). RNA was purified with chloroform extraction, and precipitated with isopropyl alcohol and 5 µl 20 mg/ml glycogen (Roche). The extracted RNA was spiked‐in with 4SU‐labeled S2 RNA and was further fragmented by base hydrolysis in 0.2 M NaOH on ice for 15 min, neutralized by adding 1× volume of 1 M Tris‐HCl pH 6.8 and precipitated with isopropyl alcohol. Biotinylation reaction of 4SU‐labeled RNA was carried out in a total volume of 250 μL, containing 100 μg total RNA, 10 mM HEPES (pH 7.5), 1 mM EDTA, and 5 μg MTSEA biotin‐XX (Biotium) dissolved in DMF (final concentration of DMF = 20%) at room temperature for 30 min. After biotinylation, excess biotin reagents were removed by extraction with chloroform and phase lock gel. RNA supernatant was precipitated with a 1:10 volume of 5 M NaCl and an equal volume of isopropyl alcohol. The RNA pellet was resuspended in 200 μl RNase‐free water. After denaturation of RNA samples at 65°C for 5 min followed by rapid cooling on ice for 5 min, biotinylated RNA was separated from non‐labeled RNA using 50 µl MyOne streptavidin C1 Dynabeads. Streptavidin beads were incubated with RNA samples for 15 min with rotation at room temperature. Beads were washed three times with wash buffer (10 mM Tris‐HCl, pH 7.4, 1 mM EDTA, 1 M NaCl, 0.1% Tween 20) at room temperature followed by one step wash with wash buffer at 65°C. 4SU‐RNA was eluted from streptavidin beads with 100 µl freshly prepared 100 mM dithiothreitol (DTT) followed by a second elution with an additional 100 µl 5 min later. RNA was purified using the SPRI beads and used for RNA‐seq library preparation. Libraries were made with the NEBNext Ultra RNA Library Prep Kit for Illumina and subjected to Illumina sequencing. TT‐seq reads were aligned to the human genome (UCSC hg38). Alignments were processed with HISAT2, allowing only uniquely mapping reads with up to three mismatches within the 150 bp read. The resulting reads were normalized to total reads aligned (reads per million, r.p.m) for each strand with deeptools 3.3.0. The read counts across each gene were counted with HT‐seq with the union mode, and Deseq2 was used to perform the differentially gene expression analysis.

### 2.14 Analysis of mRNA half‐life

For 4SU‐TT‐seq, all sequencing data have been deposited in GSE143994. Raw reads of 4SU‐TT‐seq, quality control, and adapter removing were performed by fastp, then clean reads were mapped to genome reference (hg38) and dm6 drosophila melanogaster via hisat2. Half‐life values, we first used heseq‐count to counting reads that mapped to gene exons with ensemble human annotation file (GRCh38.96) and ensemble drosophila melanogaster annotation file(BDGP6.22.96), then R packages RUVSeq and DESeq2 was used to handle spike‐ins and normalization. The half‐life time of RNA was calculated by the formula: t_1/2_ = −t_L_ * ln(2)/ln(1–R) (where t_1/2_ represents half‐life, t_L_ represents 4SU labeled time, and R represents normalized counts ratio of nascent RNA and total RNA).^31^ To compare half‐life of m^6^A‐modified gene in wild type and *YTHDF1* deleted cells, 10 m^6^A‐modified genes were selected randomly from wild type cells and *YTHDF1* deleted cells respectively to calculate the average half‐life, and then *p*‐value was calculated by Wilcoxon Signed‐Rank Test after 10,000 iterations. Actinomycin D and qPCR were used to test the half‐life changes of individual mRNA. The primers used in this research are listed in Table S2.

### 2.15 Protein purification

The pET28a‐His‐EGFP‐AGO2 or pET28a‐His‐mCherry‐YTHDF1 plasmids were transformed into bacterial strain *E*. *coli* BL21 for protein production. A single bacteria colony from each transformation was selected, inoculated in liquid Luria‐Bertani (LB) medium, and grew at 37°C overnight while being shaken at 220–250 rpm. When the OD_600_ value of bacterial culture reached 0.8, IPTG was added to the culture medium at a final concentration of 0.24 mg/ml, and the bacteria were grown at 16°C for another 24 h. The bacteria cells were harvested by centrifugation at 5000 *g* for 30 min at 4°C and frozen at −80°C until use. The cell pellet was sonicated under with 3‐sec on, 4‐sec off with 40% input (45% amplitude) at 4°C in lysis buffer, and the mixture was centrifuged at 10,000 g for 30 min at 4°C. The supernatant was transferred into a dialysis bag and concentrate with PEG6000. The recombinant proteins were purified using Ni‐NTA SefinoseTM Resin (C600033‐0100; Sangon Biotech) according to the manufacturer's instructions. Ultrafiltration centrifuge (UFC505096; Millipore) was used to concentrate the purified proteins. The purified proteins were then treated with RNase A and DNase I at 37°C for 10‐15 min  and, finally, purified again using Ni‐NTA SefinoseTM Resin. The protein concentration was measured by the BCA Protein Assay Kit (C503021, Sangon Biotech).

### 2.16 *In vitro* phase separation

For protein only: AGO2‐EGFP or YTHDF1‐mCherry protein solution was diluted with PBS or PBS containing 0.1–0.2 mg/ml BSA to make a concentration gradient and PEG6000 (8074911000, sigma) was added to the solution at a final concentration of 5%–15% (M/V). For Protein and RNA^m6A^: AGO2‐EGFP or YTHDF1‐mCherry solution and RNA^m6A^ (Concentration gradient) were all diluted in PBS. After incubation at room temperature for 5–10 min, PEG6000 was added to the solution at a final concentration of 5%–15% (M/V). If the peptide is prone to phase separation, the solution is expected to turn cloudy or opaque immediately at room temperature. The liquid droplets were examined with a fluorescence microscope or laser scanning confocal microscope.

### 2.17 Fluorescence recovery after photobleach (FRAP) analysis

Fluorescence recovery after photobleach experiments in HEK293 cells were performed on Leica laser scanning confocal microscope with a 100× oil immersion objective. The intensity of the fluorescent signal was controlled in the detection range by adjusting the laser power, digital gain, and offset. For the green channel, bleaching was conducted by a 488 nm line from an argon laser at 70%–80% intensity with 10–40 iterations. For the red channel, bleaching was conducted by a 561 nm line from an argon laser at 70%–80% intensity with 10–40 iterations. In the focal‐bleach experiment, roughly half of the particles were bleached or fully photobleached. The distribution of the fluorescence within the photo‐manipulated particle was determined over time.

## 3 RESULTS

### 3.1 *YTHDF1* knock out caused RNA patches deposited in cytoplasm

**FIGURE 1:**
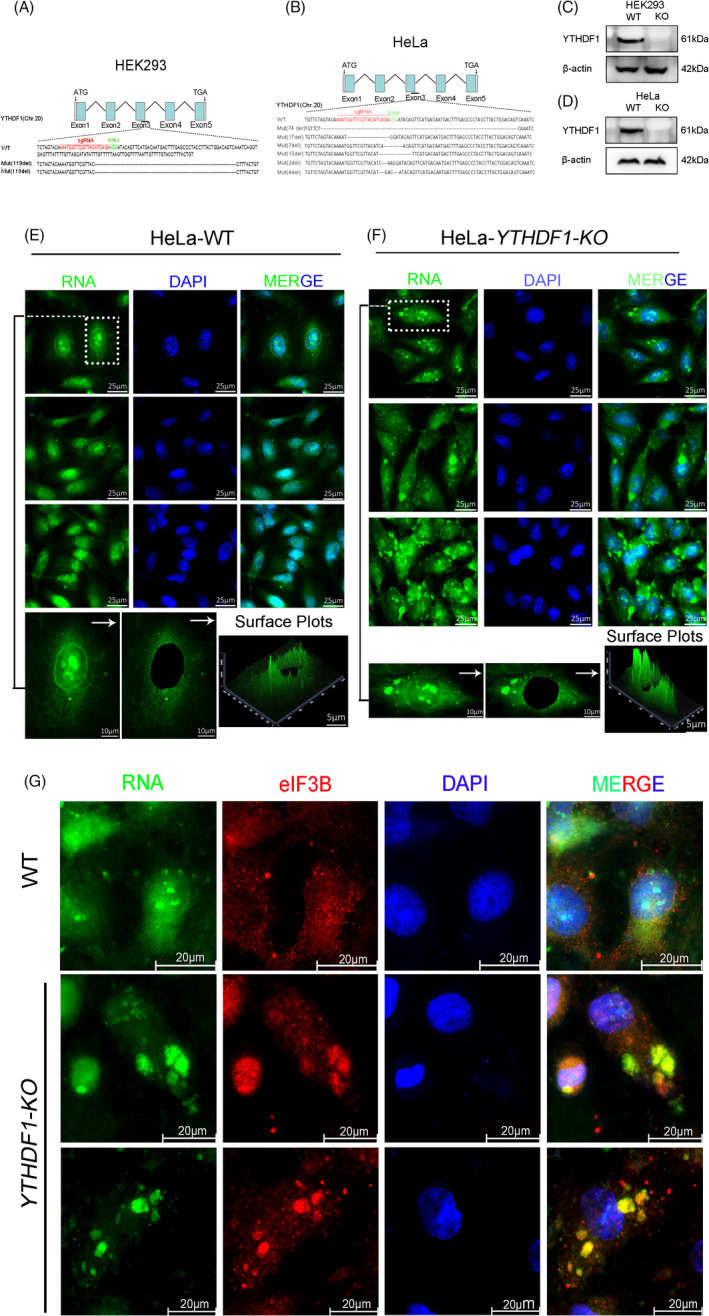
Deletion of *YTHDF1* caused RNA abnormal deposition. (A and B) YTHDF1 was silenced by CRISPR‐Cas9 in HEK293 (A) and HeLa (B) cell lines. Mutations in each allele were shown. (C and D) Western blot analysis of YTHDF1 knockdown in HEK293 (C) and HeLa cell lines (D), WT is “wild type” cells and KO is “*YTHDF1*‐*KO*” cells. (E and F) RNA stained with SYTO RNA‐select dye in HeLa WT (E) and *YTHDF1*‐*KO* (F) cell lines. The bottom image is magnification of the boxed area with 3D fluorescence intensity. G, Fluorescence imaging showed co‐localization of eIF3B and RNA in HeLa cell lines (WT and *YTHDF1*‐*KO*)

To characterize the role of YTHDF1 in mRNA metabolism, we established *YTHDF1* knockout cell lines with CRISPR‐Cas9 in HEK293 and HeLa cell lines (Figure 1A,B). Western blot analysis showed that we successfully knocked out the expression of YTHDF1 in HEK293 and HeLa cell lines (Figure 1C,D). RNA staining in HeLa cells (WT) showed uniformly distributed RNA with small particles aggregation (Figure 1E), whereas massive RNA patches were found in cytoplasm of *YTHDF1* deficient HeLa cells (Figure 1F). Consistently, 3D imaging results showed uneven distribution and significantly increased density of the RNA patches in *YTHDF1* deficient cells. Previous study was reported that YTHDF1 is suggested to promote translation of targeting transcripts in HeLa cells.^7^ To explore whether those RNA patches preserved translational activity, we co‐stained eIF3B and RNA in WT and *YTHDF1*‐*KO* cell lines. The results showed that RNA patches were co‐localized with eIF3B, it is suggested that the RNA patches have translation initiation capabilities (Figure 1G).

### 3.2 YTHDF1 promotes mRNA degradation

**FIGURE 2:**
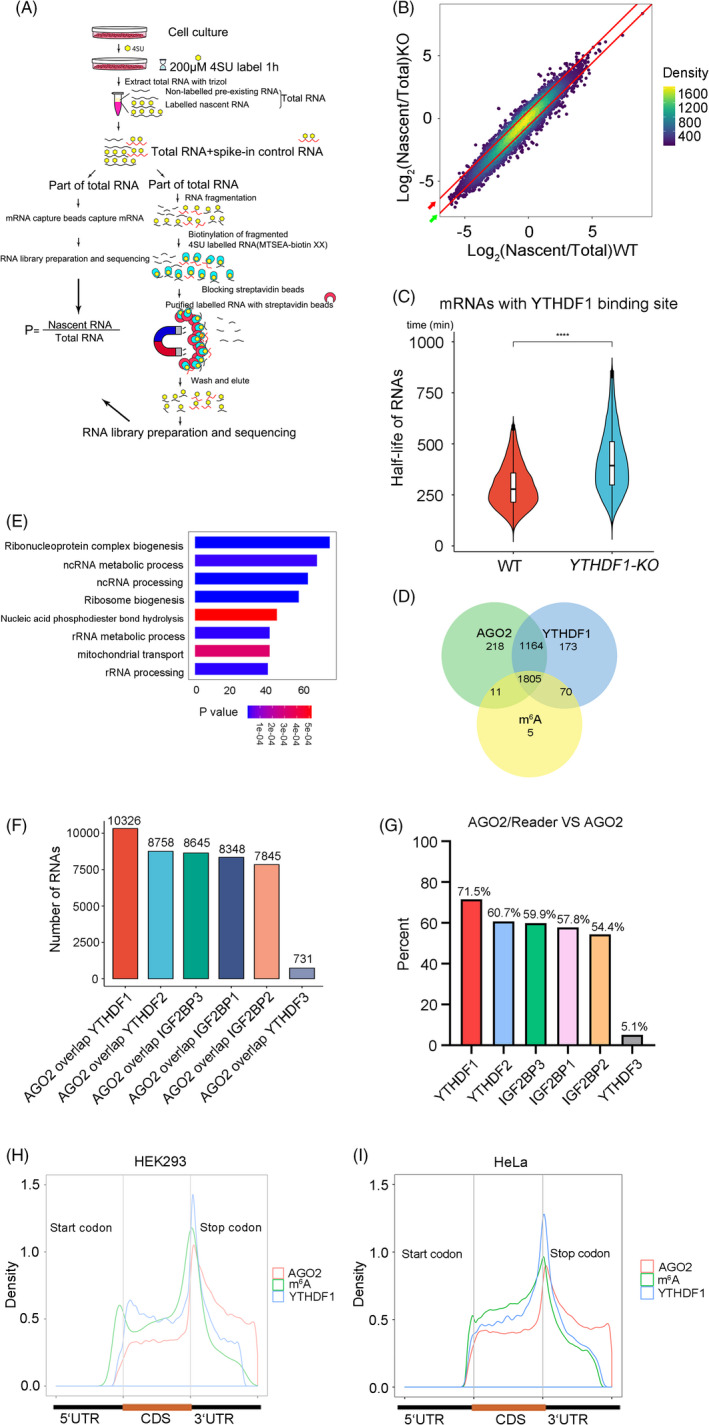
YTHDF1 regulates mRNA degradation. (A) Schematic diagram of the 4SU‐TT‐seq experiment. (B) Scatter core density diagram of stability change of RNAs in WT and *YTHDF1* deleted HEK293 cell lines. The red bars are the dividing line of half‐life changed. Half‐life prolonged 1.5 times (Red arrow), Half‐life shorten 1.5 times (Green arrow). There were totally of 3759 mRNAs have been found half‐life prolonged in our 4SU‐TT‐seq, in this half‐life prolonged group, 1507 mRNAs half‐life prolonged at least 1.5 times. (C) Half‐life change of mRNAs (KO/WT > 1) with YTHDF1 binding sites in HEK293 (WT and *YTHDF1*‐*KO*) cell lines. *p < 0.05, **p < 0.01, ***p < 0.001, ****p < 0.0001. (D) Venn diagram showed the overlap of half‐life prolonged mRNAs (KO/WT>1) related to m^6^A, YTHDF1 and AGO2. (E) GO analysis of genes that at least 1.5 times prolonged half‐life in *YTHDF1*‐*KO* HEK293 cells. (F) Overlap of RNAs bound m^6^A readers and RNAs bound AGO2. (G) The percentage of readers‐binding mRNAs were overlapped with AGO2 binding mRNAs. “AGO2/Reader” represent mRNAs with AGO2 and m^6^A reader binding site. “AGO2” represent mRNAs with AGO2 binding site. (H and I) Distribution of m^6^A modification and YTHDF1, AGO2 binding sites on mRNA transcripts in HEK293 (H) and HeLa (I) cell lines

To evaluate the RNA dynamics, we performed 4SU‐TT‐seq in WT and *YTHDF1*‐*KO* HEK293 cell lines (Figure 2A). After the 1‐hour exposure to the nucleoside analog 4‐thiouridine (4SU), newly transcribed RNAs were labeled by 4SU, which were isolated and sequenced. The sequencing results revealed that many mRNAs in *YTHDF1* deficient cells were more stable than WT cells (Figure 2B). The half‐life of mRNAs with YTHDF1 binding sites showed a significant increase in *YTHDF1*‐*KO* cells (Figure 2C). A total of 3759 mRNAs with prolonged half‐life have been found in our 4SU‐TT‐seq. In this group, 3446 mRNAs are related to YTHDF1/AGO2/m^6^A, and only 313 mRNAs have no YTHDF1/AGO2 binding sites or m^6^A modification. Furthermore, the Venn diagram showed mRNAs with prolonged half‐life (KO/WT > 1), which over 92% YTHDF1‐bound mRNAs were also occupied by AGO2 (Figure 2D). Gene enrichment analysis showed that those mRNAs were enriched in terms of ribonucleoprotein complex biogenesis, ncRNA metabolic process, and other important pathways (Figure 2E).

Previous study shows that m^6^A Peaks are enriched at the microRNA target sites,^31^ so we hypothesize that YTHDF1 promotes mRNA degradation may be related to miRNA. The miRISCs contain the Argonaute (AGO) family protein AGO2, which plays important roles in the function of miRNA.^20^, ^21^ In line with the aforementioned findings, we observed that m^6^A readers (YTHDF1/2/3 and IGF2BP1/2/3) and AGO2 had the largest number of common targeting mRNAs (Figure 2F,G) and YTHDF1 showed most significant related. Public CLIP‐seq data showed that YTHDF1 binding sites shared distribution pattern with m^6^A modification sites and AGO2 binding sites in HEK293 and HeLa cell lines (Figure 2H,I). These results suggest that YTHDF1 may regulate mRNA degradation with AGO2.

### 3.3 YTHDF1 interacts and co‐localize with AGO2 in P‐body

**FIGURE 3:**
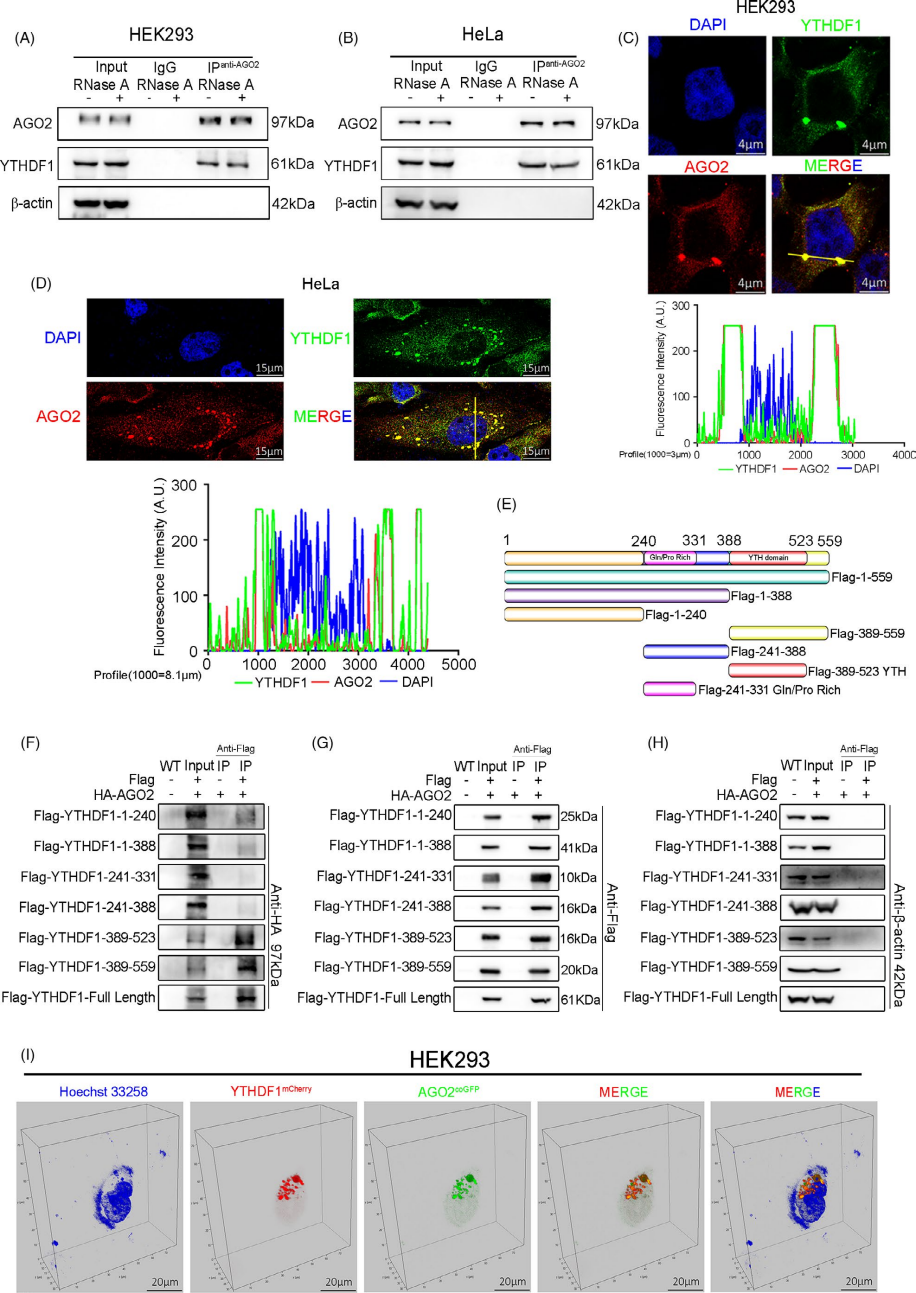
YTHDF1 interacts with AGO2 through YTH domain. (A and B) Co‐immunoprecipitation (Co‐IP) and western blot had shown the binding of YTHDF1 and AGO2 in HEK293 (A) and HeLa (B) cell lines. (C and D) Immunofluorescence and laser confocal detection of co‐localization of YTHDF1 and AGO2 in HEK293 (C) and HeLa (D) cell lines. The graph below showed the fluorescence intensity peaks along the line. (E) Schematic diagram of human YTHDF1 and the fragments used in (F–H). (F–H) Co‐IP in lentivirus (YTHDF1 tagged with FLAG and AGO2 tagged with HA) infected HEK293 cells and western blot tested the HA (F), FLAG (G), and β‐actin (H). (I) Laser confocal images showed the co‐localization of YTHDF1^mCherry^ and AGO2^coGFP^ *in vivo*

**FIGURE 4:**
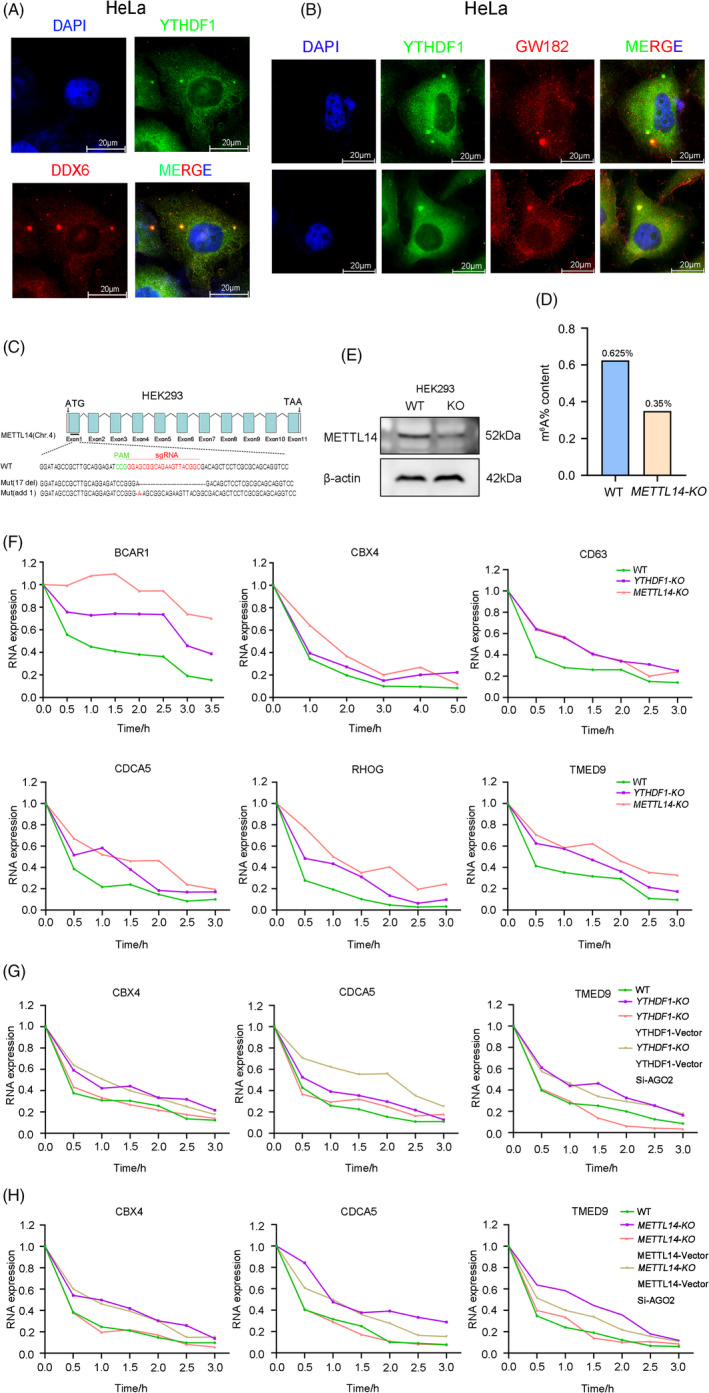
YTHDF1 regulates mRNA degradation through AGO2. (A) Immunofluorescence imaging detected co‐localization of YTHDF1 and DDX6 in HeLa cells. (B) Immunofluorescence imaging detected co‐localization of YTHDF1 and GW182 in HeLa cells. (C) *METTL14* was silenced by CRISPR‐Cas9 in HEK293 cells. Mutations in each allele were shown. (D) Content of m^6^A modification in HEK293 WT and *METTL14*‐*KO* cell lines. (E) Western blot analysis of METTL14 expresion in WT and *METTL14*‐*KO* HEK293 cell lines. (F) Half‐life change of the BCAR1, CBX4, CD63, CDCA5, RHOG, TMED9 by actinomycin D (10 μg/ml) inhibition in HEK293 cell lines (WT, *YTHDF1*‐*KO*, *METTL14*‐*KO*). (G) Half‐life change of the CBX4 CDCA5 TMED9 by actinomycin D (10 μg/ml) inhibition in HEK293 cell lines (WT, *YTHDF1*‐*KO*). YTHDF1‐Vector is represented overexpression of YTHDF1. Si‐AGO2 is represented knock down expression of AGO2 by siRNA. (H) Half‐life change of the CBX4, CDCA5, TMED9 by actinomycin D (10 μg/ml) inhibition in HEK293 cell lines (WT, *METTL14*‐*KO*). METTL14‐Vector is represented overexpression of METTL14

To confirm the interaction between YTHDF1 and AGO2, we first conducted co‐immunoprecipitation (Co‐IP) experiments and found that YTHDF1 could interact with AGO2 in RNA independent manner (Figure 3A,B). In addition, immunofluorescence analysis revealed that YTHDF1 could co‐localize with AGO2 (Figure 3C,D). To characterize the interaction domain of YTHDF1 and AGO2, we overexpressed fragmented and full length of YTHDF1 with FLAG‐tag (Figure 3E) as well as HA tagged full‐length AGO2 for Co‐IP. Western blot analysis showed that aa1‐240 and aa1‐388 showed weak signal with AGO2, aa241‐331, and aa241‐388 had no signal with AGO2, whereas aa389‐523, aa389‐559, and full length YTHDF1 exhibited strong signal with AGO2 (Figure 3F–H). These results indicated that YTHDF1 could interact with AGO2 through the 389–523 domain (YTH domain). To observe co‐localization of YTHDF1 and AGO2 *in vivo*, we performed 3D image reconstruction of whole cells and found physical co‐localization of YTHDF1 and AGO2 (Figure 3I). Since AGO2 is an important component of P‐body.^19^, ^20^ We hypothesize that YTHDF1 could co‐localize with AGO2 in P‐body. In order to obtain the precise localization of YTHDF1, we performed immunofluorescence with another two P‐body marker proteins (GW182 and DDX6) and found co‐localization of YTHDF1 and DDX6/GW182 (Figure 4A,B). Collectively, our data strongly support an interaction between YTHDF1 and AGO2, and YTHDF1 could localize in P‐body with AGO2.

To further confirm that YTHDF1 promotes mRNA degradation, we measured the mRNA decay rate after transcriptional inhibition with actinomycin D treatment in WT, *YTHDF1* (Figure 1A) or *METTL14* knock out cell lines (Figure 4C). We established *METTL14* knockout (*METTL14*‐*KO*) cell line with CRISPR‐Cas9 in HEK293 cells. Western blot analysis showed that we significantly knock down the METTL14 expression in HEK293 cells (Figure 4E). Our results showed that the half‐life of mRNAs was significantly extended after *YTHDF1* or *METTL14* deletion (Figure 4F). Then, we used *YTHDF1*‐*KO* cell line to overexpress YTHDF1 and knock down (siRNA) AGO2. We found that YTHDF1 overexpression promoted RNA degradation, while si‐AGO2 treatment inhibited this recuperation effect (Figure 4G). These results indicated that YTHDF1 likely regulates mRNA degradation through AGO2. We next sought to determine whether m^6^A modification is involved in this process. Knockout of *METTL14* significantly decreased m^6^A levels (Figure 4D) and extended the half‐life of the selected mRNAs (Figure 4F). Rescue of METTL14 expression in *METTL14* knockout cells caused the half‐life of the selected mRNAs similar to WT level. Notably, AGO2 knockdown could inhibit the recuperation effect of METTL14 re‐expression (Figure 4H). Together, these data indicate that the YTHDF1 promotes mRNA degradation through interacting with AGO2, and m^6^A modification involved in this process.

### 3.4 Both YTHDF1 and AGO2 can activate phase separation *in vitro*

**FIGURE 5:**
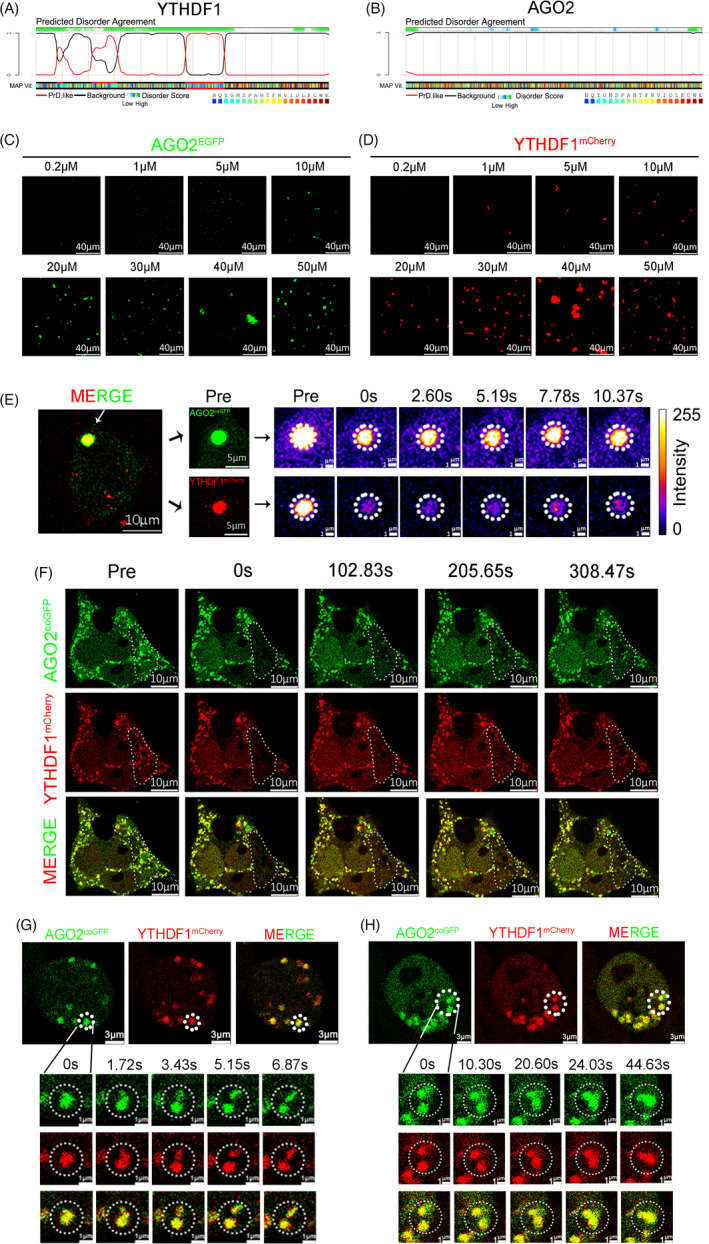
Phase separation of YTHDF1 and AGO2. (A and B) Distribution of PrLD domain and disorder sequences in YTHDF1 (A) and AGO2 (B). (C) AGO2^EGFP^ (AGO2 labeled with EGFP) underwent phase separation *in vitro* at different concentrations from 0.2 to 50 μM in 150 mM NaCl. (D) YTHDF1^mCherry^ (YTHDF1 labeled with mCherry) underwent phase separation *in vitro* at different concentrations from 0.2 to 50 μM in 150 mM NaCl. (E) FRAP of partial photo bleached YTHDF1^mCherry^ and AGO2^coGFP^ co‐localized droplets in HEK293 cells. (F) FRAP of YTHDF1^mCherry^ and AGO2^coGFP^ co‐localized droplets in HEK293 cells. (G and H) Diffusion (G) and fusion (H) events of YTHDF1^mCherry^ and AGO2^coGFP^ co‐localized droplets in HEK293 cells

YTHDF1 had Prion‐like domain (PrLD) and multiple disorder sequences (Figure 5A), which were found to be drivers of phase separation.^18^, ^32^ This observation is consistent with previous reports showing that YTHDF1 can undergo phase separation.^33^, ^34^, ^35^ In contrast, AGO2 had only a few weak disorder sequences and no PrLD domain (Figure 5B), although AGO2 was reported to aggregate miRISCs formation through LLPS.^16^ To gain more insight into the role of YTHDF1 and AGO2 in phase separation, we performed both *in vivo* and *in vitro* assays. *E*. *coli* was used to over‐express AGO2^EGFP^ and YTHDF1^mCherry^. We found that both AGO2^EGFP^ and YTHDF1^mCherry^ could undergo phase separation alone *in vitro* (Figure 5C,D), and the ability of AGO2 to drive phase separation appears to be weaker than YTHDF1. Fluorescence recovery after photobleaching (FRAP) is often performed on the droplets as an assessment of their dynamics. FRAP results showed that the P‐body fluorescence foci labeled with AGO2^coGFP^ and YTHDF1^mCherry^ were able to recover after bleaching (Figure 5E,F). Additionally, we also found that these liquid droplets could undergo diffusion and fusion *in vivo* (Figure 5G,H). These data suggest that YTHDF1 and AGO2 co‐localized P‐body is formed by phase separation.

### 3.5 YTHDF1 is more important in driving YTHDF1/AGO2 complex LLPS *in vitro*

**FIGURE 6:**
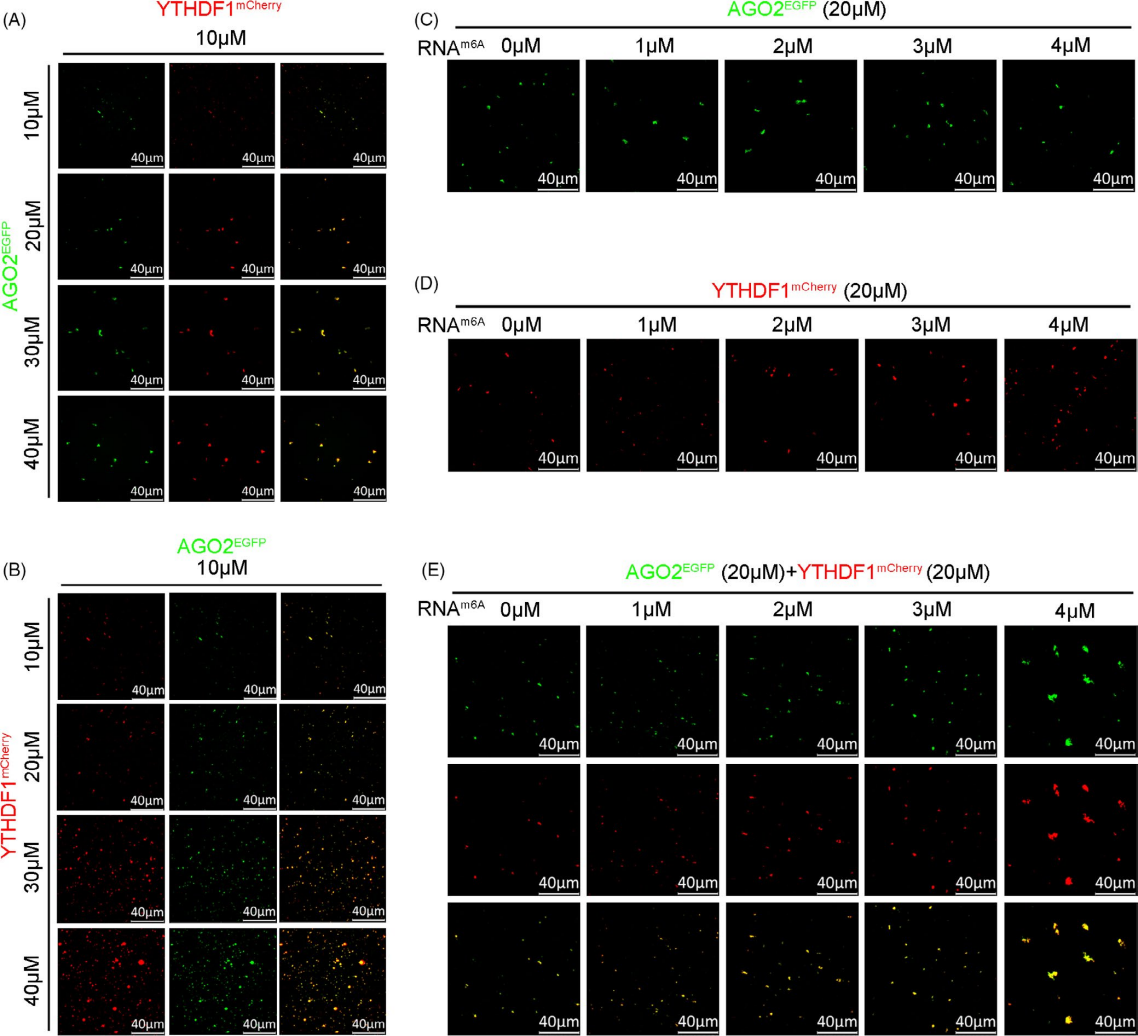
YTHDF1 driving the phase separation of YTHDF1/AGO2 complex. (A) AGO2^EGFP^ (10–40 μM) slightly increased the LLPS in the presence of YTHDF1^mCherry^ (10 μM) in 150 mM NaCl. (B) YTHDF1^mCherry^ (10–40 μM) increased the LLPS significantly in the presence of AGO2^EGFP^ (10 μM) in 150 mM NaCl. (C) RNA^m6a^ (1–4 μM) did not significantly increase the LLPS of AGO2^EGFP^ (20 μM) in 150 mM NaCl. (D) RNA^m6A^ (1–4 μM) increased the LLPS of YTHDF1^mCherry^ (20 μM) significantly in 150 mM NaCl. (E) RNA^m6A^ (1–4 μM) increased the LLPS of solutions containing AGO2^EGFP^ (20 µM) and YTHDF1^mCherry^ (20 µM) in 150 mM NaCl

To determine whether YTHDF1 or AGO2 plays a more critical role in the formation of P‐body, we performed phase separation *in vitro*. We found that in the YTHDF1^mCherry^ and AGO2^EGFP^ mixed solution, increasing the concentration of AGO2^EGFP^ had little effect on driven LLPS (Figure 6A). In contrast, raising the concentration of YTHDF1^mCherry^ substantially increased solution LLPS, in the YTHDF1^mCherry^ and AGO2^EGFP^ mixed solution (Figure 6B). In addition, a higher concentration of RNA^m6A^ (A synthetic RNA with four m^6^A modification sites) did not significantly effect on driven LLPS when solution only with AGO2^EGFP^ (Figure 6C) but largely increased LLPS in the presence of YTHDF1^mCherry^ (Figure 6D). When RNA^m6A^, AGO2, and YTHDF1^mCherry^ were mixed, increased concentration of RNA^m6A^ could markedly increase LLPS of solution (Figure 6E). Taken together, our data suggest that YTHDF1, rather than AGO2, that is likely a more important factor in driven LLPS‐process, and m^6^A modification may participate in this process.

### 3.6 Knock out of *YTHDF1* impairs P‐body formation *in vivo*

**FIGURE 7:**
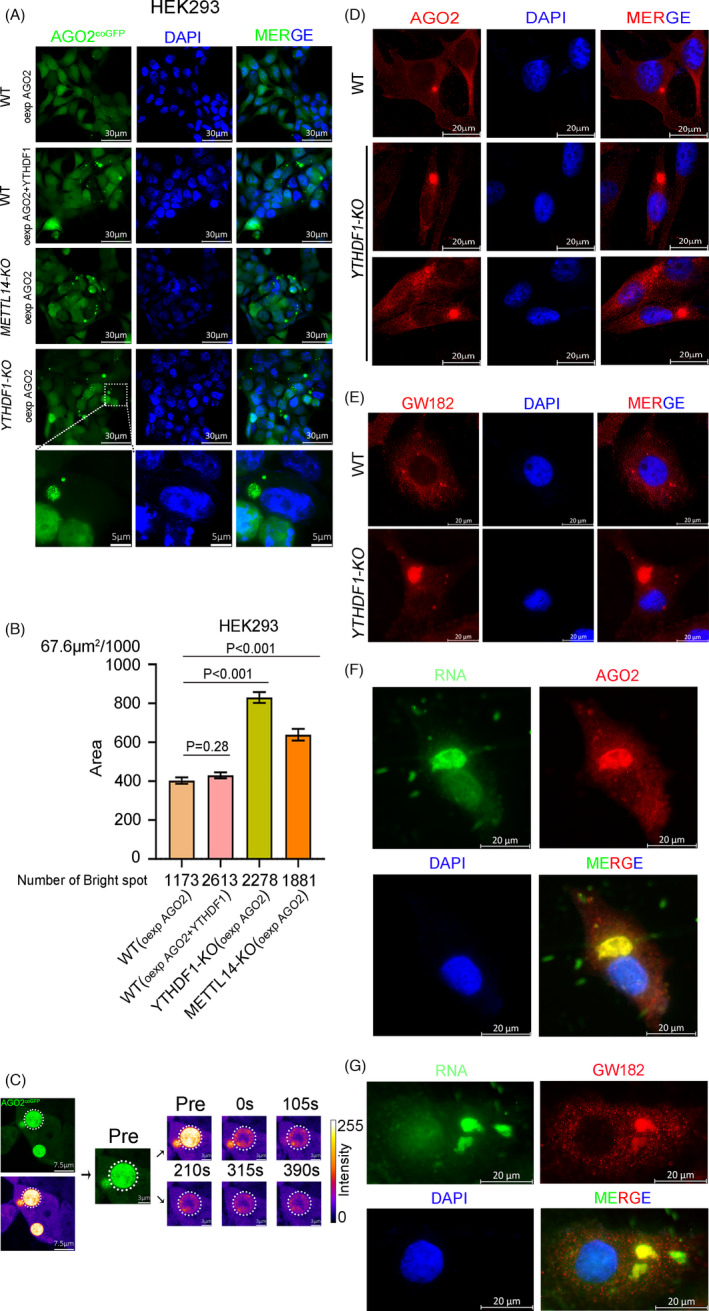
Deletion of *YTHDF1* impairs the P‐body formation. (A) Fluorescence microscope detection of AGO2^coGFP^ foci in HEK293 cell lines (WT, *YTHDF1*‐*KO*, *METTL14*‐*KO*), “oexp” is represented overexpress. (B) Area of each AGO2^coGFP^ foci in HEK293 cell lines (WT, *YTHDF1*‐*KO*, *METTL14*‐*KO*). (C) FRAP of partial photo bleached AGO2^coGFP^ foci in *YTHDF1*‐*KO* HEK293 cells. (D and E) Immunofluorescence of AGO2 (D) and GW182 (E) in HeLa cell lines (WT and *YTHDF1*‐*KO*). (F and G) Fluorescence co‐staining of RNA dye and anti‐AGO2 (F)/GW182 (G) antibodies in *YTHDF1*‐*KO* HeLa cells

To further explore the impact of YTHDF1 on P‐body formation, we established *YTHDF1* deletion (HEK293*^YTHDF1^* ^‐^ *^KO^*) (Figure 1A) and *METTL14* deletion (HEK293*^METTL14^* ^‐^ *^KO^*) cell lines (Figure 4C). We further generated other stable overexpression cell lines: HEK293 (overexpress AGO2^coGFP^), HEK293 (overexpress AGO2^coGFP^+YTHDF1^mCherry^), HEK293*^YTHDF1^* ^‐^ *^KO^* (overexpress AGO2^coGFP^), and HEK293*^METTL14^* ^‐^ *^KO^* (overexpress AGO2^coGFP^). Interestingly, the AGO2^coGFP^ foci in WT cells were uniformly fluorescent spots, whereas in the *YTHDF1* or *METTL14* knock out cell lines, many large and hollow AGO2^coGFP^ foci were observed. The results indicated abnormal formation of AGO2^coGFP^ foci (Figure 7A,B). We performed FRAP in HEK293*^YTHDF1^* ^‐^ *^KO^* (overexpress AGO2^coGFP^) and found that the large and hollow fluorescent foci failed to restore (Figure 7C). These results indicated that deletion of *YTHDF1* leaded to the conversion of AGO2 liquid droplets to gels/solids. Furthermore, we explored the state of endogenous AGO2 after *YTHDF1* deletion by immunofluorescence experiments. The results showed that compared with the WT cells, the size of AGO2 foci significantly increased after *YTHDF1* deletion, which is consistent with the findings in HEK293*^YTHDF1^* ^‐^ *^KO^* (overexpress AGO2^coGFP^) and HEK293*^METTL14^* ^‐^ *^KO^* (overexpress AGO2^coGFP^) (Figure 7D). Additionally, we used the P‐body marker GW182 to confirm the defect in P‐body formation by *YTHDF1* deletion. The results showed that the GW182 foci size showed increased which is similar to the results of AGO2 (Figure 7E). Taken together, our data demonstrate that deletion of *YTHDF1* increases the aggregation of AGO2 and causes abnormal P‐body formation.

As we found *YTHDF1* deletion led to the deposition of RNA patches in cytoplasm (Figure 1F) and impaired P‐body formation (Figure 7A–C), we co‐stained those RNA patches with AGO2/GW182 in *YTHDF1*‐*KO* cells. The results showed that the RNA patches and AGO2/GW182 were co‐localized (Figure 7F,G), indicated that the RNA patches were been able to localizing into P‐body, although the P‐body is gels/solids but not liquid character.

**FIGURE 8:**
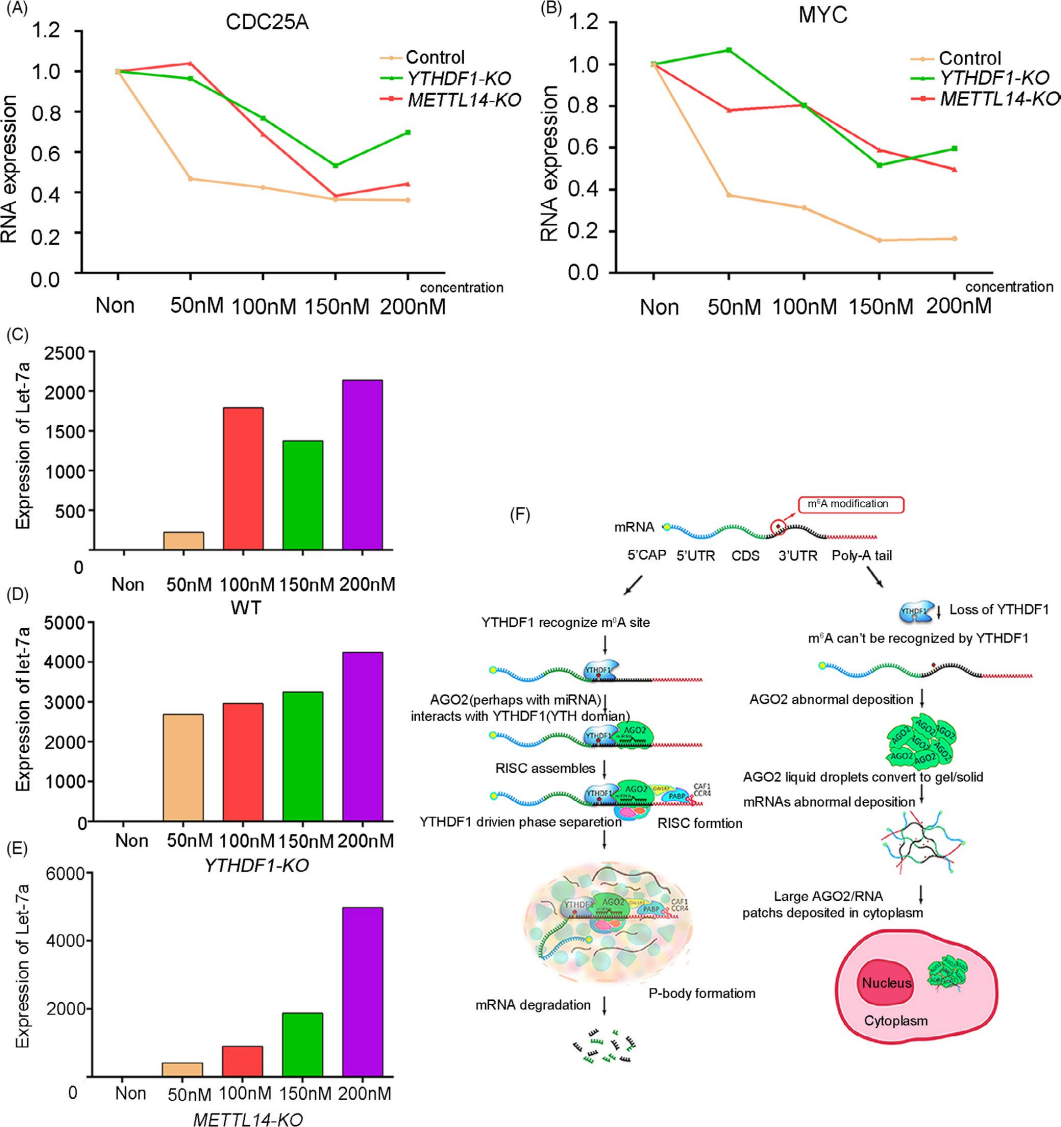
miRNA may participate in the process of YTHDF1‐AGO2 caused mRNAs degradation. (A and B) Detection of targeting genes CDC25A (A) and MYC (B) expression by qPCR in HEK293 (WT, *YTHDF1*‐*KO*, *METTL14*‐*KO*) cells lines transfected with let‐7a‐5p. (C–E) Detection of let‐7a‐5p expression by qPCR in WT (C), *YTHDF1*‐*KO* (D), *METTL14*‐*KO* (E) HEK293 cell lines. (F) Working model: YTHDF1 interacts with AGO2 (AGO2 perhaps along with miRNA) through YTH domain, then undergoes LLPS to aggregate more components of miRISCs, leading to P‐body formation for mRNA degradation. Deficiency of YTHDF1 disrupts the interaction between YTHDF1 and AGO2, leads to the conversion of AGO2 liquid droplets to gels/solids and substantially increases the mRNA stability

Previous studies reported that AGO2 is an important component of miRISCs and plays important roles in the function of miRNA.^19^, ^20^, ^21^ We found that YTHDF1 could interact with AGO2 and co‐localize in P‐body. So, whether miRNA participates in the process of mRNA degradation driven by YTHFD1. We choose let‐7 as the representative miRNA to confirm our hypothesis.^36^, ^37^ We successfully overexpressed let‐7 in HEK293 cells (Figure 8C–E), and qPCR results showed that the deletion of *YTHDF1* impede degradation of targeting mRNAs of let‐7 (Figure 8A,B). Similar results were obtained with the deletion of *METTL14* (Figure 8A and 8B). These data suggest that miRNA may participate in the process of m^6^A‐YTHDF1‐AGO2 degradation mRNAs.

## 4 DISCUSSION

The m^6^A modifications are enriched near stop codons and 3’UTRs,^38^ where AGO2 recognition sites are heavily distributed in 3’UTRs.^22^ This phenomenon implies a potential correlation between the m^6^A modification and miRNA‐AGO2 mediated gene regulation. In our research, we demonstrate that YTHDF1 through YTH domain interacts with AGO2 (AGO2 perhaps along with miRNA), then undergoes LLPS to aggregate more components of miRISCs, leading to P‐body formation for mRNA degradation. Notably, our data show that YTHDF1, rather than AGO2, is the leading factor to drive LLPS. Deficiency of YTHDF1 disrupts the interaction between YTHDF1 and AGO2, leads to the conversion of AGO2 liquid droplets to gels/solids and substantially increases the mRNA stability (Figure 8F). This mechanism raises a new sight for the mRNA regulation of YTHDF1.

The YTH‐domain‐containing proteins are involved in regulation of mRNA stability.^1^, ^39^ For example, YTHDF2 can promote the degradation of mRNAs and is localized in P‐body,^5^ with the ability to drive phase separation.^33^, ^34^ In our study, we found that YTHDF1 could interact with AGO2 through the YTH domain, but YTHDF2 and YTHDF3 also contained YTH domain, and YTHDF1/2/3 were reported with ability of driving LLPS.^33^ This interesting finding prompted us to explore whether all YTH‐domain‐containing proteins participate in this process.

In addition, previous studies showed that YTHDF1 is related to mRNA translation.^7^ To clarify whether these RNA patches are functional (translationally active) or nonfunctional (dormant or degradation intermediate) in *YTHDF1* deletion cells. We found that these RNA patches could co‐localize with eIF3B (Figure 1G), indicated that they may preserve functional translation initiation capabilities. We are not sure whether the translation efficiency is changed or not of RNAs in these patches compared with normal situation. Therefore, we speculate that there may be a balance between translation and degradation, yet the specific molecular mechanism needs further investigation.

In this study, we found YTHDF1 could interact with AGO2 through the YTH domain. Thus, it is worth investigating whether YTHDF1 affects AGO2 binding to its targeting mRNA. To verifying this speculation, we conducted AGO2‐CLIP assay. Unfortunately, the reproducibility of the experimental results is poor so reliable conclusion was not achieved. In our results, we found that the RNA patches and AGO2/GW182 were co‐localized (Figure 7F,G), indicating that these RNAs could bind to AGO2/GW182. Whether binding efficiency was affected or not is undetermined. YTHDF1 recognizes m^6^A modification, interacts with AGO2, and then undergoes phase separation to drive P‐body formation. In this process, we don’t have solid data to prove whether YTHDF1 affects AGO2 binding to targeting mRNA, Further investigation is necessary to validate our observation and speculation.

## 5 CONCLUSIONS

Overall, YTHDF1 promotes mRNA degradation via YTHDF1‐AGO2 interaction and phase separation. Our study provides new insights into the mRNA stability regulation of YTHDF1. These findings shed light on important roles of m^6^A in many physiological and pathological processes, such as tumor formation,^39^ embryo development,^40^ meiosis,^41^, ^42^ stem cell differentiation,^43^, ^44^ and DNA damage repair.^45^

## CONFLICT OF INTEREST

The authors declare that they have no conflict of interest.

## AUTHOR CONTRIBUTIONS

Mengcheng Luo, Chunjiang He, and Jiong Li designed the project and wrote the manuscript. Jiong Li carried out most of the experiments. Xin Dong and Xiangfei Mei performed most of the bioinformatics analysis. Kaiwei Liang and Honghong Wang performed 4SU‐TT‐Seq. Ke Chen supervised all the analysis and revised the manuscript. Jianjun Chen revised the manuscript. Qi Sun and Zhen Chen helped establishing cell lines. Rongyu zhang, Liuping chang, Rong Liu, Zongwen Tian, and Cong Liu participated in writing the manuscript. Yating Xu and Zhe Yang helped figure preparation. We thank Guohong Li (the Institute of Biophysics of the Chinese Academy of Sciences in China) for advice. We thank Dr. Chao Peng and Yue Yin (the Mass Spectrometry System at the National Facility for Protein Science in Shanghai (NFPS), Zhangjiang Lab, SARI, China) for data collection and analysis. We thank Fang Tao and Ellison Joe Horacek for editing.

## Supporting information

Tables S1 and S2 (In table S1， we added a new information of the antibody, the revised TableS1 was attached in the attachments)

## Data Availability

All data needed to evaluate the conclusions in the manuscript are present in the main figures and the Supplementary Materials. Additional related data are available upon request from the authors. For 4SU‐TT‐seq, all sequencing data had been deposited in GSE143994.
