# Supplementary material for: YTHDF1 promotes mRNA degradation via YTHDF1‐AGO2 interaction and phase separation
Source: Cell Prolif. 2021 Nov 25;55(1):e13157. doi: 10.1111/cpr.13157 (PMC8780909; doi:10.1111/cpr.13157)
Supplement: Supplementary file 1 — Tables S1 and S2 (In table S1， we added a new information of the antibody, the revised TableS1 was attached in the attachments) [file CPR-55-e13157-s001.docx]

| **Table S1. Antibody for WB detection** | | | |
| --- | --- | --- | --- |
| Antibody Name | Company | Catalog | Dilution |
| Anti-DDX6 | Invitrogen | Cat# PA5-18478 | 1 in 300 (IF) |
| Anti-TNRC6A | Abnova | Cat# H00027327 | 1 in 200 (IF) |
| Anti-ACTB | Abclonal | Cat# AC004 | 1 in 5000（WB） |
| Anti-AGO2 | Abnova | Cat# H00027161-M01 | 1 in 200 (IF)  1 in 1000 (WB) |
| Anti-AGO2 | Abcam | Cat# ab215746 | 1 in 1000 (WB) |
| Anti-YTHDF1 | Abclonal | Cat# A13260 | 1 in 1000 (WB) |
| Anti-YTHDF1 | Abcam | Cat# ab99080 | 1 in 1000 (WB) |
| Anti-YTHDF1 | Invitrogen | Cat# PA5-89122 | 1 in 200 (IF) |
| Anti-METTL14 | Abclonal | Cat# A8530 | 1 in 1000 (WB) |
| Anti-METTL14 | Abcam | Cat# ab98166 | 1 in 1000 (WB) |
| Anti-HA | Siama | Cat# H6908 | 1 in 1000 (WB) |
| Anti-HA | Abclonal | Cat# AE036 | 1 in 1000 (WB) |
| Anti-HA | Abclonal | Cat# AE008 | 1 in 1000 (WB) |
| Anti-FLAG | Siama | Cat# F1804 | 1 in 1000 (WB) |
| Anti-FLAG | Abclonal | Cat# AE004 | 1 in 1000 (WB) |
| Anti-FLAG | Abclonal | Cat# AE005 | 1 in 1000 (WB) |
| Donkey Anti-Goat IgG H&L (Alexa Fluor® 594) | Abcam | Cat# ab150132 | 1 in 300 (IF) |
| Goat Anti-Rabbit IgG H&L (Alexa Fluor® 488) | Abcam | Cat# ab150077 | 1 in 300 (IF) |
| Goat Anti-Rabbit IgG H&L (Alexa Fluor® 594) | Abcam | Cat# ab150080 | 1 in 300 (IF) |
| Goat Anti-Mouse IgG H&L (Alexa Fluor® 594) | Abcam | Cat# ab150116 | 1 in 300 (IF) |
| Goat Anti-Mouse IgG H&L (Alexa Fluor® 488) | Abcam | Cat# ab150133 | 1 in 300 (IF) |
| HRP Goat Anti-Rabbit IgG (H+L) | Abclonal | Cat# AS014 | 1 in 5000（WB） |
| HRP Goat Anti-Mouse IgG (H+L) | Abclonal | Cat# AS003 | 1 in 5000（WB） |

| **Table S2. Primers of qPCR detection and vector construction** | | |
| --- | --- | --- |
| Primer Name | Sequence | Usage |
| YTHDF1-F | CGCCATATGGCGATGTCGGCCACCAGCGTGGAC | Recombinant plasmid (pET28a-mcherry) |
| YTHDF1-R | CGCGGATCCGCGTTGTTTGTTTCGACTCTG |  |
| YTHDF1-F | CCGGAATTCCGGATGTCGGCCACCAGCGTGGAC | Recombinant plasmid (plVX-EF1α-mCherry) |
| YTHDF1-R | CGCGGATCCGCGTTGTTTGTTTCGACTCTG |  |
| AGO2-F | CGCGGATCCGCGATGTACTCGGGAGCCGGCCCC | Recombinant plasmid (pET28a-EGFP) |
| AGO2-R | CCCAAGCTTGGGAGCAAAGTACATGGTGCG |  |
| AGO2-F | CCGGAATTCATGTACTCGGGAGCCGGCCC | Recombinant plasmid (pcDH-CMV-coGFP) |
| AGO2-R | CGCGGATCCAGCAAAGTACATGGTGCGCA |  |
| METTL14-F | CCCAAGCTTGGGATGGATAGCCGCTTGCAGGA | Recombinant plasmid (pCMV-FLAG) |
| METTL14-R | CGCGGATCCGCGTTATCGAGGTGGAAAGCCAC |  |
| MYC-F | ACAACCGAAAATGCACCAGC | qPCR |
| MYC-R | GTCGTTTCCGCAACAAGTCC |  |
| CDC25A-F | GGCAAGCGTGTCATTGTTGT | qPCR |
| CDC25A-R | AGGGTAGTGGAGTTTGGGGT |  |
| CBX4-F | GCTGCTGATCGCCTTCCA | qPCR |
| CBX4-R | GCTTGGCACGGTTGTCAGT |  |
| BCAR1-F | AAGACCAGCAGCATCCAGTC | qPCR |
| BCAR1-R | TCCCCTGTAGGTGGACGTAG |  |
| CD63-F | GTTGCCAGTGGTCATCAT | qPCR |
| CD63-R | ACAGAAAGATGGCAAACG |  |
| RHOG-F | GTGCCTGCTCATCTGCTACA | qPCR |
| RHOG-R | CACACCTCTGGATGCCACTT |  |
| TMED9-F | AGGCATGCTGAGAGTTCACC | qPCR |
| TMED9-R | GTCTGCAGAATGGACCACCA |  |
| CDCA5-F | GCATCCTCCCTGAAATCTG | qPCR |
| CDCA5-R | TCAGGGTTCGGCACAGGA |  |
| Let-7a-5p-F | TGAGGTAGTAGGTTGTAT | qPCR |
| U6-F | CTCGCTTCGGCAGCACA | qPCR |
| U6-R | AACGCTTCACGAATTTGCGT | qPCR |
